# Spontaneous bilayer wrapping of virus particles by a phospholipid Langmuir monolayer

**DOI:** 10.1140/epje/s10189-023-00366-8

**Published:** 2023-12-05

**Authors:** J. F. Torres-Salgado, M. V. Villagrana-Escareño, A. L. Duran-Meza, X. F. Segovia-Gonzalez, R. D. Cadena-Nava, W. M. Gelbart, C. M. Knobler, J. Ruiz-García

**Affiliations:** 1 Biological Physics Laboratory, Institute of Physics, Universidad Autónoma de San Luis Potosí, San Luis/dF Potosí, 78000 San Luis Potosí, México; 2 Present Address: Present Address: Center of Nanosciences and Nanotechnology-UNAM, Km 107 Carretera Tijuana-Ensenada, 22800 Ensenada, BC México; 3 Department of Chemistry and Biochemistry, University of California Los Angeles, Los Angeles, CA 90095-1569 USA

## Abstract

We report here the spontaneous formation of lipid-bilayer-wrapped virus particles, following the injection of “naked” virus particles into the subphase of a Langmuir trough with a liquid monolayer of lipids at its air–water interface. The virus particles are those of the well-studied cowpea chlorotic mottle virus, CCMV, which are negatively charged at the pH 6 of the subphase; the lipids are a 9:1 mix of neutral DMPC and cationic CTAB molecules. Before adding CCMV particles to the subphase we establish the mixed lipid monolayer in its liquid-expanded state at a fixed pressure (17.5 mN/m) and average area-per-molecule of (41Å^2^). Keeping the total area fixed, the surface pressure is observed to decrease at about 15 h after adding the virus particles in the subphase; by 37 h it has dropped to zero, corresponding to essentially all the lipid molecules having been removed from the air–water interface. By collecting particles from the subphase and measuring their sizes by atomic force microscopy, we show that the virus particles have been wrapped by lipid bilayers (or by two lipid bilayers). These results can be understood in terms of thermal fluctuations and electrostatic interactions driving the wrapping of the anionic virus particles by the cationic lipids.

**Graphical Abstract:**

**Figure:**
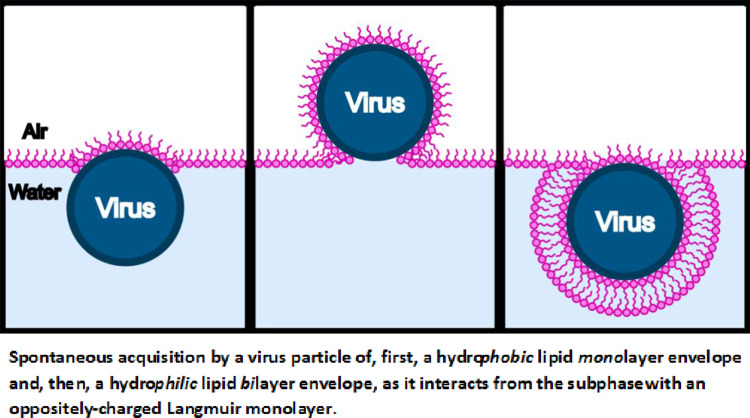
Spontaneous acquisition by a virus particle of, first, a hydrophobic lipid monolayer envelope and, then, a hydrophilic lipid bilayer envelope, as it interacts from the subphase with an oppositely charged Langmuir monolayer

## Introduction

The adsorption of small particles at liquid surfaces and interfaces has been the subject of a broad range of experimental investigations. Studies of small latex particles and quantum dots at the air/water interface have explored the physics of their organizing into foams, clusters and crystalline arrays [1–6]. The air/water interface can also trap biological molecules, such as proteins, which can then be transferred onto solid substrates by the Langmuir–Blodgett technique and be used in a variety of applications [7, 8]. The physics of adsorption of viruses at the air/water interface has also been examined [9], a topic of relevance as well to public health [10]. It is observed that viruses in the subphase diffuse to the air/water interface where they become irreversibly trapped.

A richer variety of phenomena is encountered when a lipid monolayer is present at the interface. Under those circumstances the electrostatic interaction between the particles in the subphase and the charged head groups of the amphiphile plays an important role. Investigations have been carried out on the interaction between positively charged amphiphiles and negatively charged viruses—e.g., cowpea mosaic virus [11], turnip yellow mosaic virus [12] and the procapsid of P22 bacteriophage [13]—but in each case with a subphase at a pH close to the isoelectric point of the virus so that the electrostatic interaction is weak. Under these conditions the virus forms an ordered crystal-like array beneath the monolayer of the amphiphile. Of greater relevance to biology are particle-lipid interactions at interfaces under conditions at which electrostatic interactions are more significant [14], and these are the subject of the present research.

In particular, we investigate in this work the interaction of cowpea chlorotic mottle virus (CCMV) nanoparticles with a Langmuir monolayer composed of a mixture of dimyristoylphosphatidylcholine (DMPC) and cetyltrimethyl ammonium bromide (CTAB) lipids; the DMPC headgroup is zwitterionic, while that of CTAB is positively charged. Experiments are carried out at a subphase pH of 6, for which the zeta potential of the CCMV virus is − 64 mV, corresponding to a charge of roughly −160e on each capsid [15], and at a surface pressure for which the monolayer is in the liquid-expanded (LE) phase. When viruses are introduced into the subphase with the area of the monolayer held fixed, there is a continuous decrease in the surface pressure starting at about 15 h, dropping to essentially zero by 37 h. We show that this behavior is associated with the envelopment of the particles by a bilayer of lipid and with their subsequent re-entry into the subphase. Imaging of the monolayer by Brewster-angle microscopy and characterization of particles at the surface layer and in the subphase by AFM support this interpretation.

## Experimental section

### Materials and methods

The CCMV virus was obtained from infected leaves of cowpea plants (Vigna unguiculata) as described by Lavelle, et al. [16]. The leaves were sand-milled and filtered through cheese cloth to remove debris, and then centrifuged three times to purify the virus. Following purification the virus was suspended in virus buffer, (100 mM sodium acetate and 1 mM EDTA, pH 5.0) before use. Stock solutions of DMPC (Avanti Polar Lipids, Alabaster Al, USA) and CTAB (Sigma-Aldrich, San Luis, MI, USA) were prepared in HPLC chloroform (Fermont, Monterrey, NL, MX) at concentrations of 0.3 mg/ml.

### Langmuir trough measurements

The lipids and their mixture were deposited at the air–water interface and compressed between two Teflon barriers using a Nima film balance (Nima Technology Ltd., Coventry, U.K.) The trough was filled with 600 ml of ultra-pure milli-Q water of 18.2 mΩ cm^−1^ resistivity (previously autoclaved and filtered, at a controlled temperature of 20 °C). The pH was adjusted to 6.0 by the addition of 0.01 M HCl. Brewster-angle microscopy imaging of the monolayers was performed with an Accurion Model EP4 Imaging ellipsometer (Accurion, Göttingen).

### AFM measurements

The AFM images were obtained with a Nanoscope VIII (Bruker, Billerica, MA USA), in tapping mode, suitable for soft matter samples. The scanning was performed using Bruker RTESP-300 tapping tips with a 315 Hz resonance frequency and spring constant *k* = 0.3 N/m. The peak-sample interaction force was set to 1 V, with a scanning frequency between 0.5 and 1.5 Hz. Imaging was carried out at 256, 512 and 1024 lines of resolution using a J scanner with an image range between 1 and 6 µm. Freshly cleaved mica was used as a support. Samples of particles from the interface were transferred by the Langmuir–Blodgett technique. Removal of particles from the subphase when the surface pressure was close to zero was accomplished by using a syringe with a long needle passed beneath a trough barrier and collecting about 1 ml of liquid. A few drops were then deposited on the mica substrate. The surface- and subphase-transferred films were allowed to dry in a desiccator for 12 h before being imaged.

## Results and discussion

**Fig. 1:**
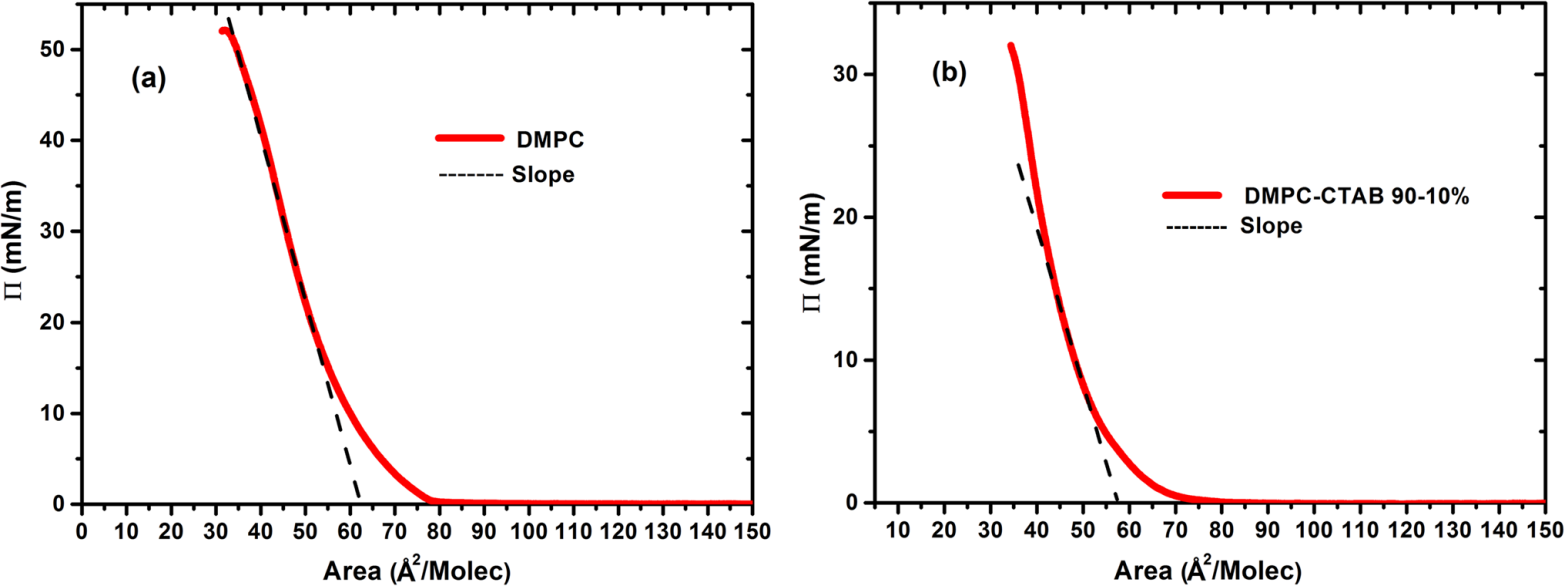
Isotherms of monolayers of DMPC and of a 90–10% DMPC-CTAB mixture, showing intersections of their slopes with the *x*-axis. The approximate values at the intersection points, corresponding to the molecular areas in the liquid-expanded phase, are ≈ 63 Å^2^ for the DMPC isotherm and ≈ 57 Å^2^ for the mixture

**Fig. 2:**
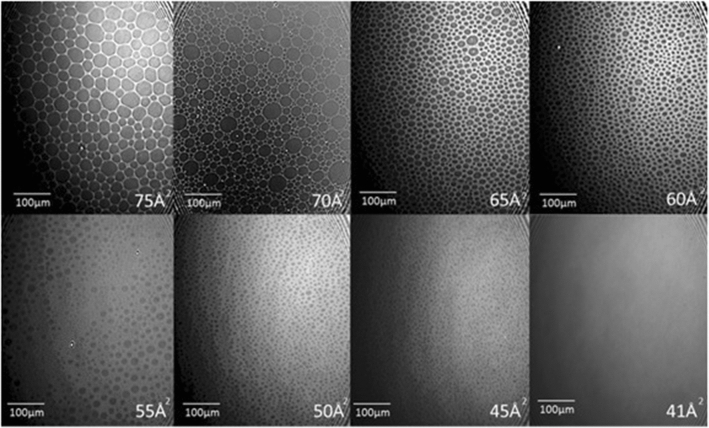
BAM images, at *T* = 20 °C and pH = 6.0, of the monolayer states of 90–10% DMPC-CTAB mixtures at different areas per molecule. The size of each image is 450 × 540 µm^2^. The mixed monolayer state evolves from two-state G-LE coexistence (Π ≈ 0 mN/m and average areas per molecule of 75 Å^2^), involving a hexagonal foam-like structure, to a homogenous single LE phase (Π ≈ 20 mN/m and an area per molecule of 41 Å.^2^)

Isotherms at 20 °C and pH 6.0 were determined for pure DMPC and for the DMPC-CTAB mixture at a compression rate of 20 cm^2^/min. Figure 1 shows a comparison of these isotherms, where we see that addition of the single-chain CTAB displaces the isotherms to a smaller area. A linear extrapolation of the isotherm slopes to **≈** zero pressure, which can be taken as the molecular area of the liquid-expanded phase at the liquid–gas coexistence, shows a value of **≈** 63 Å^2^/molecule for DMPC and of **≈** 57 Å^2^/molecule for the mixture. The phase behavior underlying the mixed-monolayer isotherm is clearly seen in the BAM images in Fig. 2, which show the progression upon compression from the two-phase gas–liquid expanded (G-LE) coexistence at hundreds of Å^2^/molecule to a homogeneous LE phase at tens of Å^2^/molecule.

**Fig. 3:**
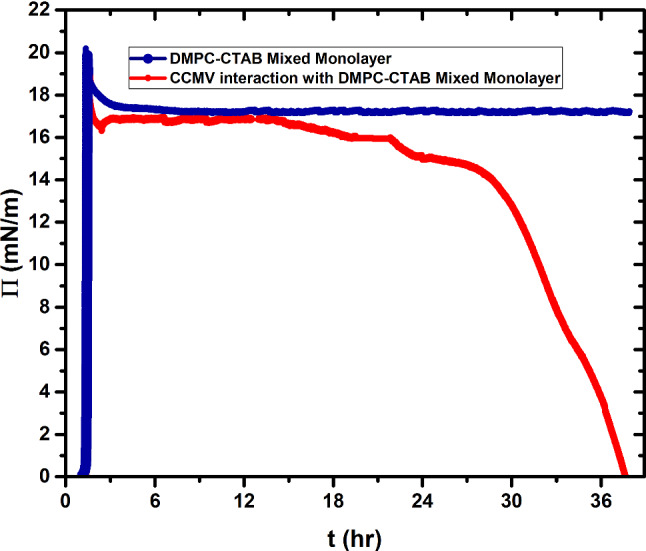
Surface pressure as a function of time. The blue curve is a plot of the pressure versus time of the DMPC-CTAB monolayer spread on the pure water subphase at pH 6. The red curve shows the behavior of the pressure when the subphase contains virus

**Fig. 4:**
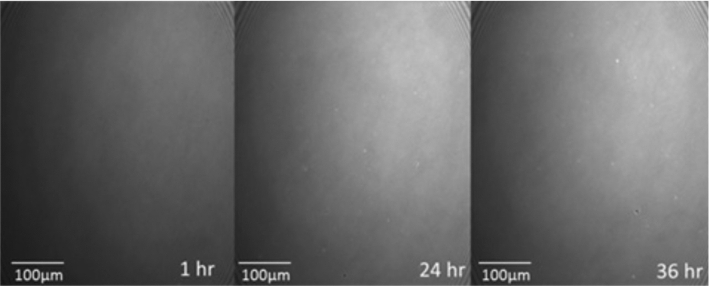
BAM images, as a function of time, of the DMPC-CTAB mixed monolayer at Π = 17.5 mN/m and area per molecule of 41 Å^2^, at *T* = 20 °C and pH = 6.0. The size of the images is 450 × 540 µm^2^. The mixed monolayer is shown to be highly stable, with no domain formation or change in phase or molecular orientation

In order to obtain the conditions for formation of a dense, stable, liquid monolayer phase of the lipid mixture, the DMPC-CTAB monolayer was compressed until the point of collapse and the compression halted until the film reached equilibrium as indicated by a stable pressure. The monolayer was then re-expanded, recompressed to a slightly higher pressure, and finally allowed to relax. As shown in Fig. 3 the pressure then remained stable at Π = 17.5 mN/m, as seen here for a period in excess of 37 h (see blue curve). Characterization of the monolayer under these conditions by BAM, see Fig. 4, shows that the monolayer remains in a one-phase region throughout this time.

**Fig. 5:**
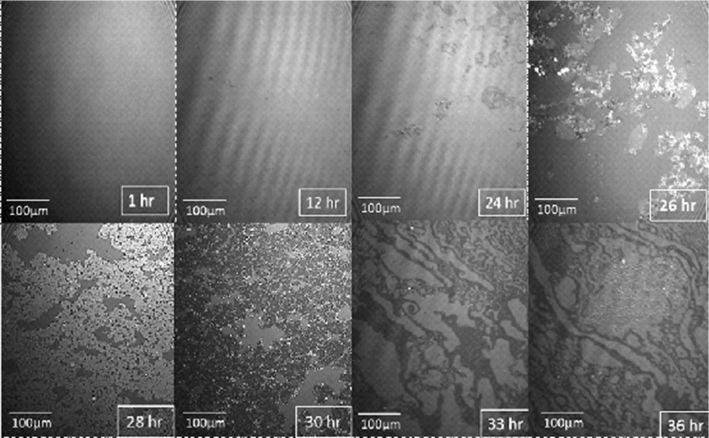
Time course of BAM images (540 × 450 µm^2^ in size) acquired during the interaction of CCMV particles with a DMPC-CTAB monolayer that has been initially equilibrated at Π = 17.5 mN/m in its LE phase at the air–water interface and then maintained at a constant area

**Fig. 6:**
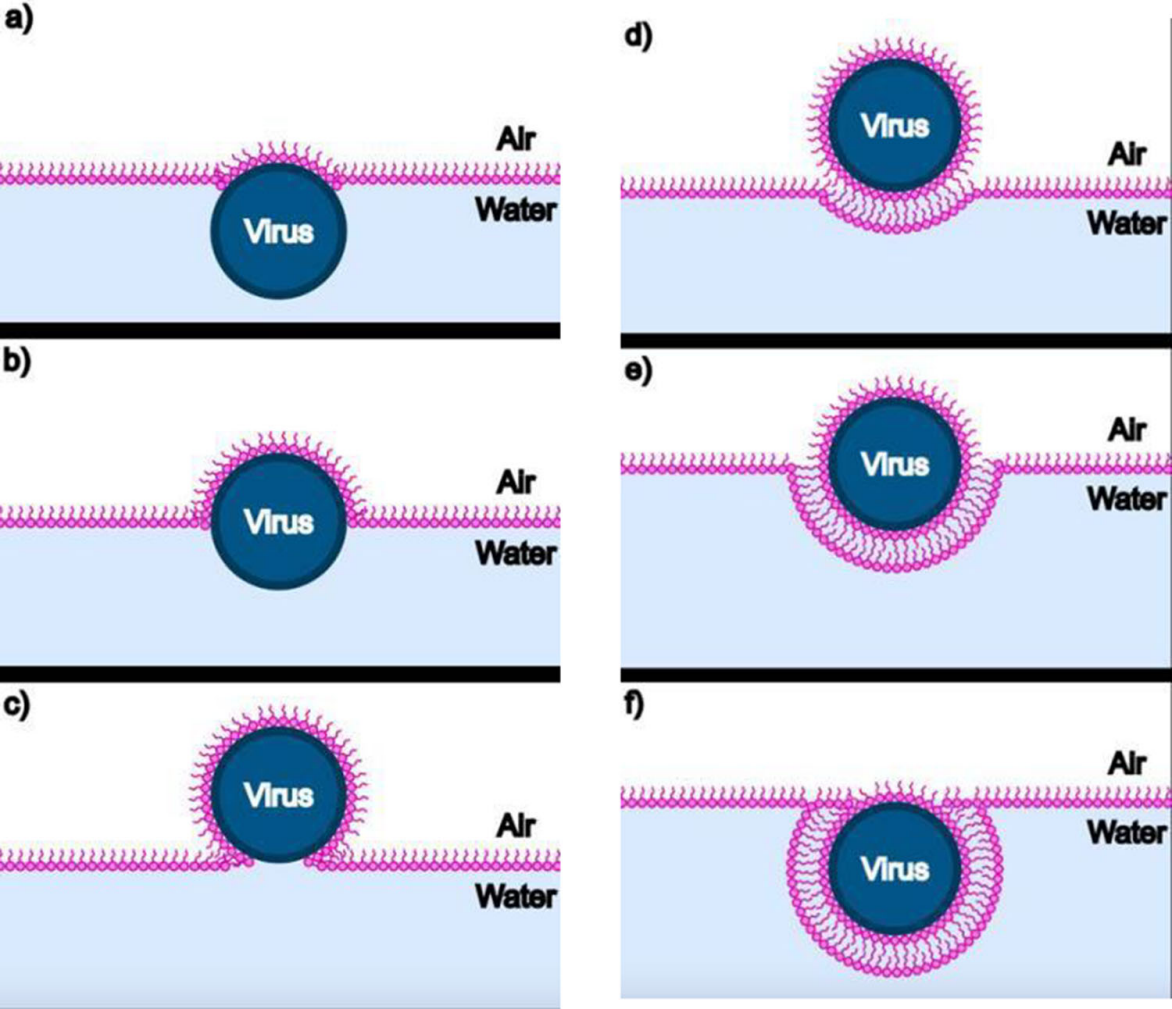
Schematic showing the acquisition at the air–water interface of a lipid monolayer envelope by a virus particle in the subphase (see **a**–**c**), and its subsequent re-entry into the subphase upon acquisition of a second lipid leaflet as it becomes fully enveloped by a lipid *bi*layer (see **d**–**f**)

Markedly different behavior is observed when 2 ml of a 0.01 mg/ml solution of CCMV particles is injected into the subphase under the same initial conditions, i.e., after establishing the mixed monolayer in its LE phase at a pressure of 17.5 mN/m and area per molecule of 41 Å^2^. As shown by the red curve in Fig. 3, the pressure remains essentially constant for about 15 h but then begins to drop, falling dramatically to zero at 37 h. Insight into this behavior is provided by BAM imaging (Fig. 5): after 12 h the initially homogeneous monolayer begins to show the presence of regions of a lower density (gas) phase, along with a small number of white dots indicative of the presence of viral particles. The size of the dots increases as the pressure drops (24 h). At 26 h, three different types of domain appear in the monolayer, identified by three unequal reflections of the p-polarized light. These domains correspond to the close-packed lipid monolayer (dark gray), to the CCMV trapped at the interface (white small dots) and, finally, to the lipids that have undergone a change in their molecular orientation. The maximum CCMV adsorption appears to occur 30 h after virus particle injection but the particles disappear very quickly (~ 33 h), leaving only elongated monolayer domains at the interface with a clear gas phase present, which remained after 37 h. This remaining film has respread and exhibits G-LE phase coexistence. Note that this behavior contrasts significantly from that when virus particles in the subphase interact with a *bare* air–water surface, in which case once the particles diffuse to the surface they remain irreversibly adsorbed [9]. It appears that electrostatic interaction between the positively charged monolayer and the negatively charged virus particles is sufficient to result, at first, in wrapping of the virus with a monolayer of lipid, leaving the particles hydrophobic and incompatible with the water subphase: see progression depicted schematically in Fig. 6a–c, and the discussion of Fig. 10 where AFM images of particles found at the air–water interface are presented and analyzed.

At later times, however, further decrease in surface pressure corresponds to return of particles to the subphase concomitant with further loss of lipid in the water–air monolayer. The only way this can happen is for the monolayer-wrapped particles to have acquired a second, “outer,” monolayer leaflet of lipid, resulting in a bilayer wrapping of particles that renders them hydrophilic again. This is illustrated schematically in Fig. 6d–f: see also Fig. 9 where AFM images of particles found in the aqueous subphase are discussed.

Because the surface area of the naked virus particle (~ 2500 nm^2^) is roughly 5000 times the area per lipid molecule (~ 0.5 nm^2^) in the monolayer, a large number (~ 5000) of lipid molecules is removed from the surface each time a particle is wrapped in a monolayer. Noting that the 2 ml of 0.01 mg/ml of CCMV solution originally injected into the subphase implies about 2 × 10^12^ virus particles, we can conclude that monolayer wrapping of them will involve removal of as many as 10^16^ lipid molecules. Formation of *bilayer*-wrapped particles more than doubles the number of lipids removed from the monolayer. And similarly for the effect of *trilayer*-wrapped and *double-bilayer*-wrapped particles (see discussion below of AFM-determined particle sizes). This accounts for a significant fraction of the total number of lipids (~ 5 × 10^16^) that were originally deposited on the water–air surface, consistent with the observed drop in surface pressure and with the smaller amount of lipid that is evident in the BAM images. Note that the progression from a to c in Fig. 6 takes many hours because it depends on “rocking” fluctuations of the virus particle at the air–water surface: gravitational forces on the virus are negligible as it rises higher above the surface—by “Brownian ratcheting”—upon acquiring a more and more complete hydrophobic leaflet of lipid.

At later times the images show that—in addition to the loss of lipids from the air–water surface—there is a concomitant *loss of particles to the subphase*, which is possible only if they have been wrapped by a second leaflet of lipids, i.e., they have been enveloped by a phospholipid bilayer and are once again hydrophilic. As depicted in d-f of Fig. 6 this process depends—like that shown in Fig. 6a–c—on “rocking” fluctuations of the virus particle that facilitate its acquisition of a more and more complete hydrophilic bilayer and its consequent “sinking”/diffusion into the water subphase.

**Fig. 7:**
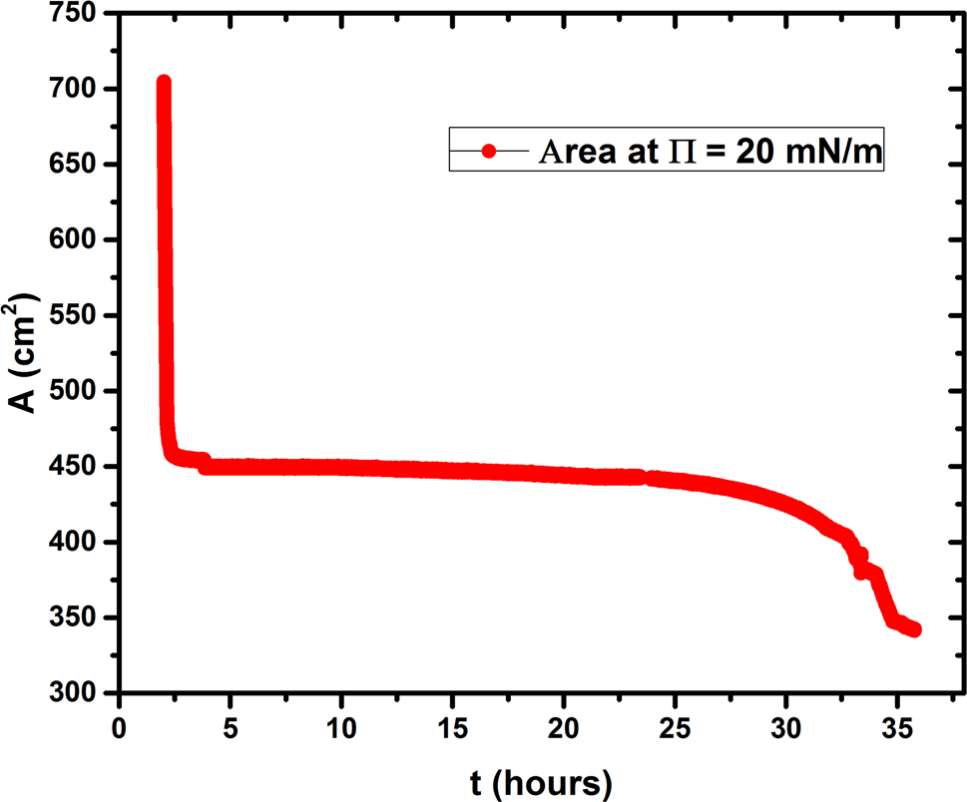
Change in monolayer area with time at fixed pressure of 20 mN/m after injection of virus into the subphase. At the start of the experiment the DMPC/DPPC mixed monolayer was spread at an area of 700 cm^2^ and compressed to 450 cm^2^ where a pressure of 20 mN/m was reached. The trough was then put in a fixed-pressure mode in which the area adjusts to maintain the pressure, and virus was injected into the subphase. Over a period of 35 h the area is seen to decrease by over 100 cm^2^

Additional proof of the loss of monolayer through interaction with the virus, and the loss of the enveloped particles into the subphase, are experiments in which the monolayer is brought into the LE phase by compression to a surface pressure 20mN/m, *and the pressure then held constant*. Unlike what is found for the case of no virus in the subphase (where the pressure remains constant without the need to adjust the surface area), significant decreases in area are necessary after virus has been injected into the subphase. The corresponding area versus time curve is shown in Fig. 7. As expected, we detect a loss of matter at the air–water interface over the period from 14 to 36 h: The monolayer surface area change (decrease) was more than 100 cm^2^. Note that this is contrast to the surface area *increase* reported by Ishitsuka, et al. [17], who injected membrane-penetrating peptides into the subphase that simply insert into the surface monolayer.

**Fig. 8:**
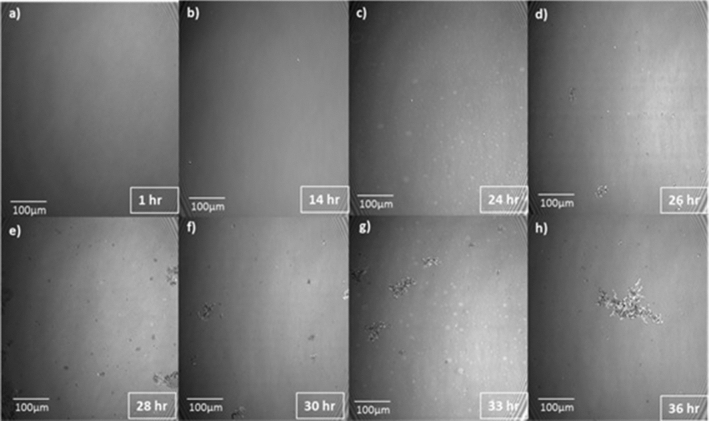
BAM images of the mixed monolayer interacting with subphase CCMV, maintained at a constant surface pressure of 20 mN /m at T = 20 °C and pH = 6.0. Surface Areas: **a** 300; **b** 297; **c** 294; **d** 286; **e** 280; **f** 275; **g** 220; and **h** 125 cm^2^. The time at which each area was reached is indicated in the bottom-right corner of the figures

In Fig. 8 the BAM images taken during the constant-pressure measurement show that the monolayer remains mostly homogeneous during the 36 h course of the experiment. Particles begin to appear around 28 h when the area occupied by the monolayer has decreased by about 10%. Some small lighter gray domains, likely the result of change in molecule orientation, appear during the period over which entrapment of CCMV particles at the interface seems to reach a maximum.

### Atomic force microscopy sample analysis

In order to further elucidate the interactions between lipids and virus particles that underlie the above results, we transferred the surface film to a mica support by the Langmuir–Blodget technique and spread samples from the subphase on mica and examined both sets of samples by AFM. In all cases the system was allowed to equilibrate for 37 h after injecting “naked” virus particles into the subphase, keeping the surface area constant.

**Fig. 9:**
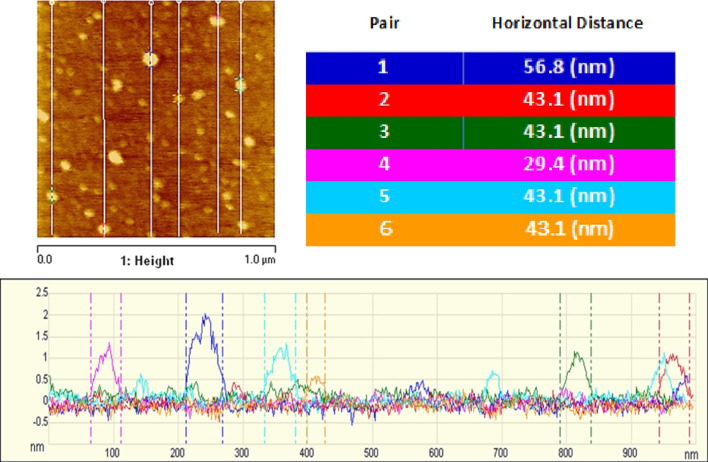
AFM scans of six particles (left), with corresponding height profile (bottom) and table of diameters (right), found in a 1.0 × 1.0 µm scanned sample taken from the water subphase

AFM measurements of the diameters of 6 particles found in the subphase showed—see Fig. 9—1 particle with diameter ~ 29 nm, 4 with diameters of ~ 43 nm, and 1 with diameter ~ 57  nm. The smallest diameter (~ 29 nm) is consistent with that of an unwrapped virus particle (~ 27 nm, from X-ray and electron microscopy [18]) plus hydration layer (~ 2 nm). The thickness of DMPC bilayers has been determined by Wong, et al. [19] from surface force apparatus studies of bilayers supported on soft polymer cushions, from which the bilayer, including a hydration surface layer, was found to be ~ 7 nm thick, in agreement with the thickness determined by Janiak, et al. [20] from X-ray diffraction. From these facts we can conclude that the ~ 43 nm particles correspond to virus capsids wrapped by a single bilayer, and the ~ 57 nm particles to double-bilayer-wrapped capsids. Note that the unwrapped particles found in the subphase have not yet found their way to the surface, whereas the singly- and doubly- bilayer-wrapped particles have returned to the subphase upon picking up the full single and double envelopes that “solubilize” them.

**Fig. 10:**
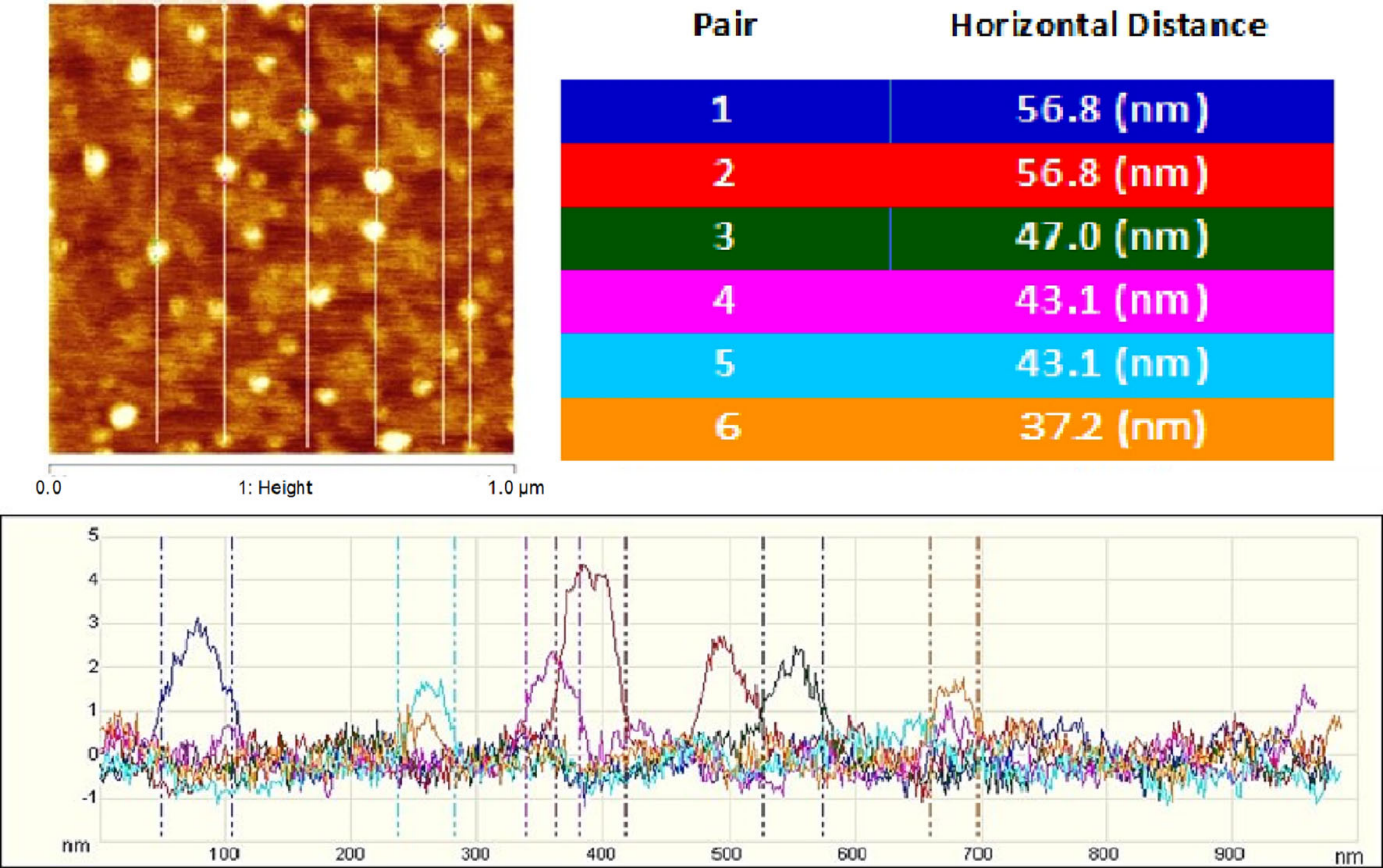
AFM images (left), and corresponding height scans (bottom) and table of diameters (right), of six particles found in a 1.0 × 1.0 µm sample taken from the air/water interface, 37 h after injecting virus particles in the subphase

Finally, AFM measurements of the sizes of 6 particles from the air–water surface show—see Fig. 10—1 particle with a diameter of ~ 37 nm corresponding to a capsid wrapped by single monolayer (~ 7 nm) and water hydration layer (~ 2 nm), 1 with a diameter of ~ 47 nm that corresponds to a *tri*-layer-wrapped particle, and 2 each with diameters of ~ 43 nm and ~ 57 nn. Note that the mono- and tri- layer particles are strongly hydrophobic and can *only* be found at the air–water surface. The ~ 43 nm and ~ 57 nn particles, on the other hand, are bilayer- and double-bilayer-wrapped particles that are hydrophilic but that *have not yet found their way into the water subphase*: They are “waiting for” the fluctuations that will allow them to diffuse out of the surface into the bulk. No measurements were made of partially wrapped particles on the surface (like those shown in Fig. 6d–f).

## Conclusions

In this work we have followed the evolution of a positively charged monolayer at the air–water interface, after it has been prepared in a liquid-expanded state and allowed to equilibrate, at constant surface área, with a subphase into which negatively charged viral capsids have been injected. After about 12 h the surface pressure is observed to decrease from its original value, accompanied by transfer of lipid into the subphase. By 36 h, the pressure has dropped to essentially zero, corresponding to a negligible number of lipids remaining at the air–water interface. Using BAM imaging of the surface, and AFM examination of particles deposited onto a mica surface after removal from the air-water interface and from the subphase, we are able to correlate this behavior with the spontaneous wrapping of virus particles from the subphase by monolayers, bilayers, triple monolayers and double bilayers of lipids. The main driving force is the electrostatic attraction between the oppositely charged lipids and capsids, supplemented by the hydrophobic attraction between inner and outer leaflets of phospholipid bilayer. In particular, we find that bare viral capsids are able to become progressively wrapped by lipid monolayer at the air–water surface, where they are “trapped” until further fluctuations allow them to acquire an “outer” monolayer leaflet and return to the subphase as hydrophilic bilayer-wrapped particles. The primary role of fluctuations here is consistent with the roughness of the surface water and lipid monolayer reported by Lu, et al. in their neutron diffraction studies [21].

The fact that a significant fraction of the virus injected into the subphase ends up as bilayer-wrapped particles suggests that the experiment we have reported here may be the cleanest and most efficient way to synthesize enveloped virus-like particles (EVLPs)—capsids surrounded by a single bilayer. All we need to do is introduce a slight excess of virus—or in vitro reconstituted virus-like particles (VLPs)—into the subphase and then wait for them to be returned to the subphase as bilayer-wrapped particles, as described above. Further, by controlling the pH of the subphase one can “tune” the sign and magnitude of the capsid charge [22] and use a mixed monolayer with appropriately opposite charge.

More explicitly we start by equilibrating a liquid-expanded phase of charged lipid monolayer at the surface, and add to the subphase a number of oppositely charged virus particles that is not quite sufficient to accommodate all of the lipid in bilayer form, namely, just less than 1 capsid per 10^4^ lipids. Keeping the area of the surface constant, many hours are needed before the particles are bilayer wrapped in the subphase because: (1) It takes hours for the particles to diffuse to the surface; and, (2) more significantly, it takes still longer for the particles and lipids at the surface to undergo the fluctuations that allow the particles to be wrapped—first by one monolayer leaflet and then by another—so that they can return in hydrophilic *bilayer*-wrapped form to the subphase. Note that “kinetic bottlenecks” arise involving single- and triple- monolayer wrapped particles that are trapped at the surface awaiting fluctuations that will impart them with the missing additional leaflet needed to be soluble in the subphase. Even bilayer and double-bilayer wrapped particles can be “stuck” in local free energy minima at the surface, waiting for fluctuations to get them started diffusing into the bulk.
